# Comment on: Benefits of tolvaptan on early dyspnea relief in patients with acute heart failure: A meta‐analysis

**DOI:** 10.1002/clc.23994

**Published:** 2023-03-02

**Authors:** Cong Yin, Juan Li, Lin Yuan

**Affiliations:** ^1^ Hubei Province Key Laboratory of Traditional Chinese Medicine Resource and Chemistry Hubei University of Chinese Medicine Wuhan Hubei China; ^2^ Hubei Provincial Key Laboratory of Occurrence and Intervention of Rheumatic Diseases Hubei Minzu University Enshi Hubei China

Dear Editor

With great interest, we read the article by Shang et al.^1^ published in August 2022 in the *Clinical Cardiology*. The authors performed a meta‐analysis and concluded that “tolvaptan treatment significantly improved patient‐assessed dyspnea early and persistently in patients with acute heart failure.” At the outset, we would like to congratulate the authors for writing an informative article with novelty. Nevertheless, we have several suggestions and queries that we would like to communicate with the authors.

First, the authors evaluate the quality of the included studies using the Detsky Quality Assessment Scale. Although this scale is used for the risk of bias assessment, the National Institutes of Health and Cochrane Library suggest a qualitative analysis of this question. The Cochrane tool has become the standard method to evaluate the risk of bias in experimental studies.^2^ Moreover, the selection of the more definite approaches, such as the GRADE (Grading of Recommendations Assessment, Development and Evaluation) approach, is highly suggested to define the level of evidence.

Second, the authors mentioned that four databases and clinicaltrials.gov were searched in this study, with no language restrictions. It would have made the results more convincing if the authors had included other databases such as Wanfang Data, Scopus, the China National Knowledge Infrastructure (CNKI), and the World Health Organization Trials Portal (ICTRP) to obtain more literature with Chinese or other language. The chances of articles in other languages being ignored may be further narrowed.

Third, the authors did not register the protocol in the International Prospective Register of Systematic Reviews (PROSPERO). Protocol registration is now recognized as the desirable approach to enhance and maintain transparency in the process and to assist in minimizing the risk of selective outcome reporting bias.^3^

Fourth, we need the detailed retrieval strategies to repeat the meta‐analysis. But the paper did not provide it. And according to the requirements of Preferred Reporting Items for Systematic reviews and Meta‐Analyses (PRISMA), detailed retrieval strategies should be provided in the article.^4^ At the same time, the PRISMA has been updated to PRISMA 2020 to reflect recent advances in systematic review methodology and terminology,^3^ we can use the latest PRISMA statement to complete systematic reviews and meta‐analyses.

In summary, we have learned the author's standardized meta‐analysis process. We hope to discuss with the authors about the above doubts. And we think that our discussion can make the conclusion of meta‐analysis more rigorous.

## CONFLICT OF INTEREST STATEMENT

The authors declare no conflict of interest.
